# Predictability of COVID-19 Hospitalizations, Intensive Care Unit Admissions, and Respiratory Assistance in Portugal: Longitudinal Cohort Study

**DOI:** 10.2196/26075

**Published:** 2021-04-28

**Authors:** André Patrício, Rafael S Costa, Rui Henriques

**Affiliations:** 1 Instituto Superior Técnico Universidade de Lisboa Lisboa Portugal; 2 LAQV-REQUIMTE NOVA School of Science and Technology Universidade NOVA de Lisboa Caparica Portugal; 3 IDMEC Instituto Superior Técnico Universidade de Lisboa Lisboa Portugal; 4 Instituto de Engenharia de Sistemas e Computadores–Investigação e Desenvolvimento Lisboa Portugal

**Keywords:** COVID-19, machine learning, intensive care admissions, respiratory assistance, predictive models, data modeling, clinical informatics

## Abstract

**Background:**

In the face of the current COVID-19 pandemic, the timely prediction of upcoming medical needs for infected individuals enables better and quicker care provision when necessary and management decisions within health care systems.

**Objective:**

This work aims to predict the medical needs (hospitalizations, intensive care unit admissions, and respiratory assistance) and survivability of individuals testing positive for SARS-CoV-2 infection in Portugal.

**Methods:**

A retrospective cohort of 38,545 infected individuals during 2020 was used. Predictions of medical needs were performed using state-of-the-art machine learning approaches at various stages of a patient’s cycle, namely, at testing (prehospitalization), at posthospitalization, and during postintensive care. A thorough optimization of state-of-the-art predictors was undertaken to assess the ability to anticipate medical needs and infection outcomes using demographic and comorbidity variables, as well as dates associated with symptom onset, testing, and hospitalization.

**Results:**

For the target cohort, 75% of hospitalization needs could be identified at the time of testing for SARS-CoV-2 infection. Over 60% of respiratory needs could be identified at the time of hospitalization. Both predictions had >50% precision.

**Conclusions:**

The conducted study pinpoints the relevance of the proposed predictive models as good candidates to support medical decisions in the Portuguese population, including both monitoring and in-hospital care decisions. A clinical decision support system is further provided to this end.

## Introduction

### Background

COVID-19 is a disease caused by the novel coronavirus SARS-CoV-2, transmissible from person to person and associated with acute respiratory complications in severe cases [1,2]. The main symptoms of patients infected are fever, cough, and fatigue; others are asymptomatic [3]. The COVID-19 pandemic presents a substantial threat to global health and has been directly responsible for many deaths. Since the first outbreak in December 2019 in Wuhan, China, the number of confirmed infected patients worldwide has exceeded 55 million cases, and nearly 1.3 million people have died from COVID-19 [4]. Current literature has shown that infected patients with specific comorbidities or preconditions (eg, hypertension, respiratory problems, diabetes) and of old age are expected to develop a more severe response to the infection and may consequently need longer hospitalizations and intensive care [5-7]. Strict social confinement measures have been implemented to decrease the COVID-19 R_0_ value (average number of individuals infected by each infected person) and guarantee the optimal use of equipment and beds at normal, continuous, and intensive care units (ICUs). However, although public health responses aim to delay the spread of the infection, several countries such as the United States, Brazil, Italy, and India have faced severe health care crises.

Without effective antiviral drugs and a vaccine, prognostic tools related to COVID-19 are required. Statistical and computational models could assist clinical staff in triaging patients at high risk for respiratory failure to better guide the allocation of medical resources. Recently, several predictive models ranging from statistical and score-based systems to more recent machine learning models have been proposed in response to COVID-19. Guan et al [8] proposed a Cox regression model to infer potential risk factors associated with serious adverse outcomes in patients with COVID-19. Univariate and multivariate logistic regression models have been used to determine risk factors associated with mortality [9]. Scoring systems have been proposed to predict COVID-19 patient mortality but are limited by small sample sizes, with a poor discriminatory ability [10-12]. Other statistical approaches have also been emerging to aid prognostics [13,14]. Complementarily, machine learning methods offer the possibility to model more complex data relationships, generally yielding powerful capabilities to predict outcomes of infectious and noninfectious diseases in medical practice [15-17]. To this end, classification and regression models have been proposed for risk stratification of patients and to screen the spread of COVID-19 [18-20]. Despite the inherent potentialities of ongoing efforts, studies in the context of COVID-19 are limited by either the size of available cohorts or the lack of a systematic comparison of different models [21-24], and generally neglect the predictability of medical needs (instead the focus is commonly placed on measurable disease factors, early detection of infection, and mortality risk prediction [25-28]). None of these studies have comprehensively targeted the Portuguese population at the present time.

### This Study

This study provides a structured view on the predictability of hospitalizations, ICU admissions, respiratory assistance needs, and survivability outcomes using a retrospective cohort encompassing individuals with a SARS-CoV-2–positive result in Portugal as of June 30, 2020.

To this end, and considering demographic, comorbidity, and care provision variables collected for the infected individuals, an assessment methodology was conducted, whereby state-of-the-art predictive models were hyperparameterized and robustly evaluated in order to assess the upper bounds on the predictive performance for each one of the targeted variables. In addition, whenever applicable, this analysis was extended toward the various stages of a patient’s cycle: prehospitalization (at the time of testing), after hospitalization, and after ICU admission.

This study offers a solid methodology for the robust assessment of the predictability guarantees of future care needs of infected individuals, contrasting with the dominant correlation-based guarantees in literature. As comparable studies demonstrated in other populations, it lays a solid ground to compare type-I and type-II predictive errors and assess population-wise differences.

## Methods

### Overview

**Figure 1:**
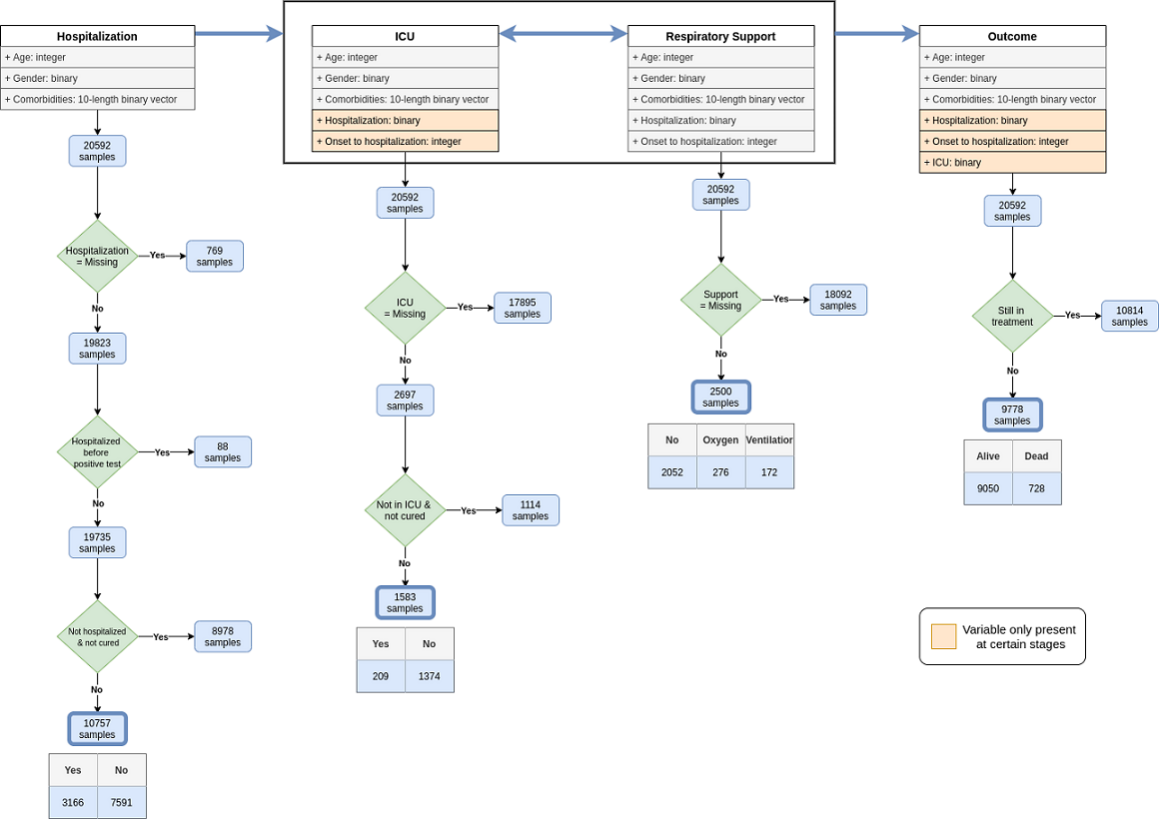
Exclusion and inclusion criteria for composing the outcome-conditional cohorts: hospitalization, respiratory assistance support, intensive care unit (ICU) admission, and survivability. Blank/unknown cells in the Direcção-Geral da Saúde data set are classified as missing values.

Complete subpopulations from the target cohort were identified for each output (Figure 1), guaranteeing the presence of all individuals undertaking the target forms of care (hospitalization, ICU admission, respiratory support) with a recovery-or-death outcome.

After the sampling and data curation steps (Figure 1), we proceeded to the optimization of data preprocessing options and classifiers’ parameterization for each of the target variables separately. To this end, we applied a nested 10-fold cross-validation assessment methodology, whereby we first create train-test partitions (outer cross-validation) to assess the performance of an optimized classification method, and within each training fold we further create train-test partitions (inner cross-validation) for hyperparameterizing the predictive model under assessment. This methodology guarantees that all observations are used to assess the final performance and prevents biases as hyperparameterization takes place within each training folds.

Within each inner train-test fold, Bayesian optimization [29] was applied to find the hyperparameters that best fit the pipeline. The optimization measures are:

- F1 score and 0.7 × recall + 0.3 × precision for binary classes. These two views generate two sets of classifiers: one that equally weights recall-and-precision views, and other that, similar to the F2 score (F_β_, where β=2), prioritizes the optimization of the true-positive rate (recall) at the cost of a lower positive predictive value (precision);
- Cohen kappa and average class recall for target variables with more than 2 classes (respiratory support).

Hospitalization, UCI admission, respiratory support, and recovery-or-death outcomes for SARS-CoV-2–infected individuals are considerably imbalanced, hence, the relevance of the placed recall-precision and multiclass recall views. In particular, considering both a balanced recall-precision optimization and recall-oriented optimization is relevant for clinical decisions. When the allocated teams have capacity to remotely monitor SARS-CoV-2–infected patients, the predictive models optimized with a schema that prioritizes recall should be pursued to guarantee that no vulnerable patient is left out. Nevertheless, when monitoring capacity is limited, greater attention to precision is necessary, and only more vulnerable patients (as suggested by the predictive models optimized with balanced recall-precision views) should be attemptively monitored.

The allowed preprocessing options are as follows: imputation of missing values using median-mode imputation, KNNImputer, or none; class balancing using subsampling, oversampling, SMOTE (Synthetic Minority Oversampling Technique), or none; and normalization of real-valued variables using standardization, scaling, or none. The selected classifiers are as follows: Bernoulli naive Bayes, Gaussian naive Bayes, k-nearest neighbors (KNN), decision tree (DT), random forest, XGBoost (XGB), logistic regression, Light Gradient Boosting Machine (LightGBM), Super Learner, and multilayer perceptron (MLP). Super Learner uses folding to hyperparameterize models and selects predictors for out-of-fold predictions from individual performance estimates per fold. In this context, Super Learner’s performance is generally coincident with the best predictive model and thus not always disclosed in the *Results* section to allow the identification of the best underlying predictors. We considered the implementations provided in the *scikit-learn* [30] and *xgboost* [31] packages in Python (Python Software Foundation). For each classifier, all supported parameters in scikit-learn were subjected to hyperparameterization. Regarding the MLP, we placed upper limits on the number of hidden layers (3) and nodes per layer (20) given the low-dimensionality nature of the target data set. The hyperparameters were subjected to a total of 50 iterations. Multimedia Appendix 1 displays the optimized parameters for the best-performing predictive models per outcome.

Differences in performance from the paired-error estimates collected per fold were statistically tested using *t* tests when estimates passed the Shapiro–Wilk normality test. When this condition was not satisfied, Wilcoxon signed-rank tests were applied.

In addition to the conducted analysis, the best predictors trained on the whole data set were made available within a clinical decision support system built using flask technology and dash facilities in Python [32], which can run as an offline web application.

### Data Source

A retrospective cohort (from March 1 to June 30, 2020) of patients with confirmed COVID-19 in Portugal was used in this study. The anonymized data set was provided by the Directorate General of Health (Direcção-Geral da Saúde, DGS), the Portuguese health authority. The gathered data, called the *covid19-DGS* database, contains information pertaining to the demographic and clinical patient characteristics as well as preexisting conditions.

Data are available upon reasonable request.

### Ethical Considerations

The COVID-19 data set is provided by the DGS under the collaborative *score4COVID* research project proposal. The tasks conducted in the *score4COVID* project were further validated by the Ethical Committee of the NOVA School of Science and Technology.

## Results

Results on the predictability of hospitalization needs, ICU admissions, respiratory assistance, and outcome of infected individuals living in Portugal, as of June 30, 2020, are discussed below.

### Cohort Characteristics

**Figure 2:**
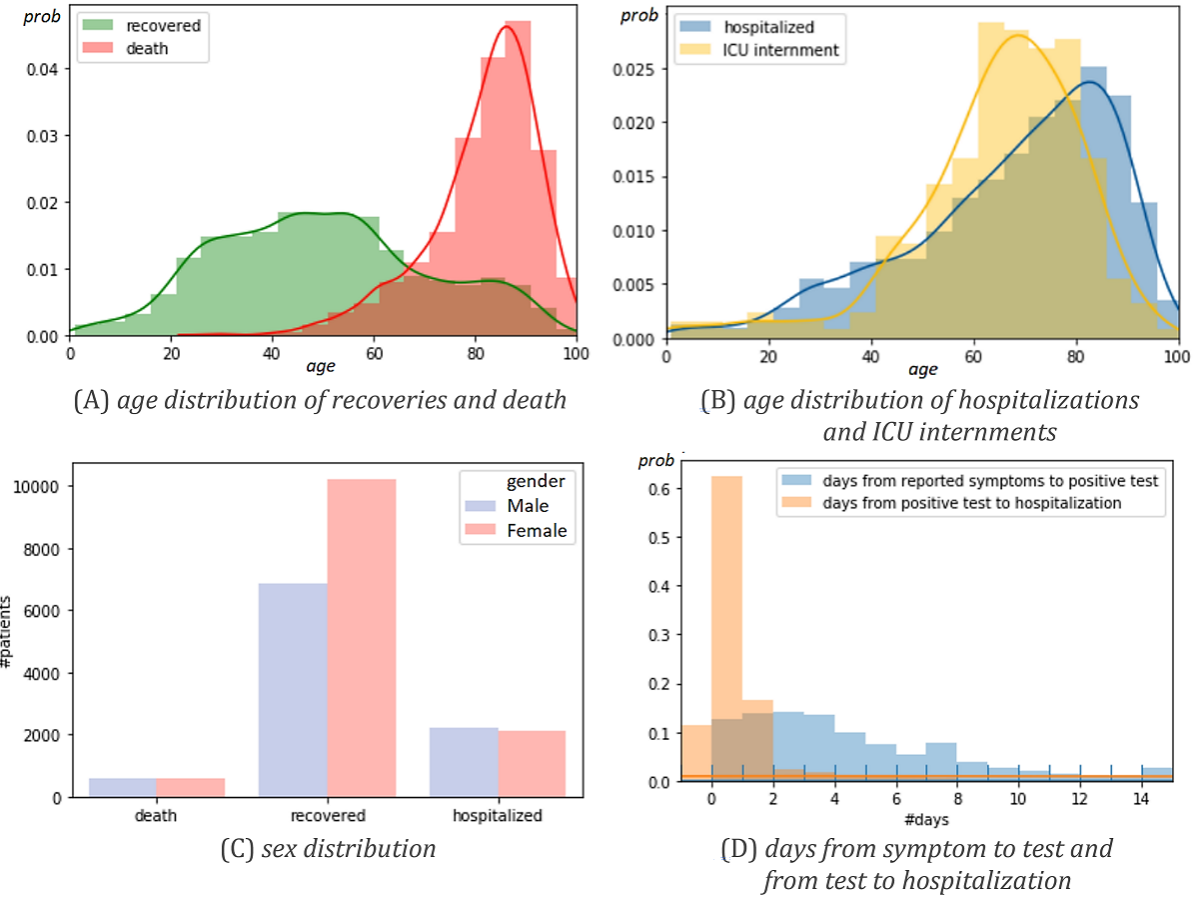
Cohort statistics: (A-C) demographic distribution of infected individuals with known outcome (death and recovery) and stage in the care life cycle (hospitalization and intensive care unit [ICU] admission); (D) average number of days between care stages (the plotted negative bin [ie, negative occurrences] corresponds to hospitalizations before SARS-CoV-2 testing).

The target cohort comprised 38,545 individuals who were SARS-CoV-2 positive: 17,046 recoveries (SARS-CoV-2 negative after positive testing) and 1155 deaths. Four individuals were excluded from the data set due to inconsistent recordings related to age and pregnancy-gender variables. Table 1 provides essential statistics. Figure 2 further describes sex and age distributions in deaths, hospitalizations, ICU admissions, and average number of days from symptom onset (traced by the public health line for COVID-19) to a positive test result and hospitalization.

Within the target population, there were 4326 hospitalizations (11.2% of population base) and 253 admissions to the ICU (5.8% of hospitalizations). Among ICU internments, there were 82 recoveries and 61 deaths. In terms of respiratory support, a total of 180 individuals undertook assisted ventilation, 292 submitted to oxygen therapy, and 9 underwent alternative modes of respiratory support such as extracorporeal membrane oxygenation.

**Figure 3:**
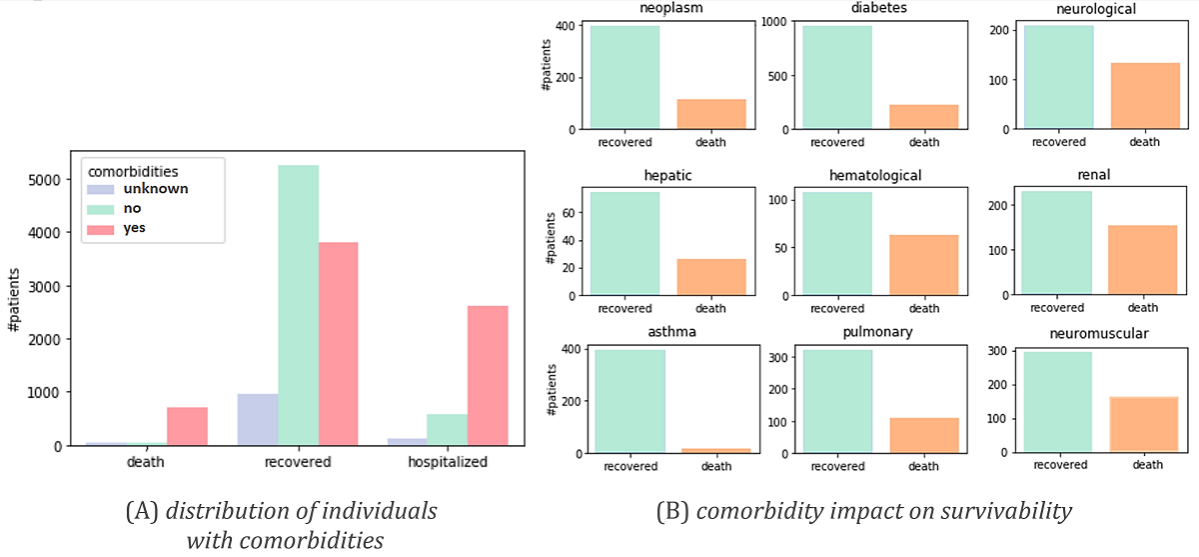
Cohort statistics: (A) distribution of individuals with one or more comorbidities among deaths, recovered cases, and hospitalizations; and (B) association between individual comorbidities and survivability outcomes.

The major classes of comorbidities monitored were neoplasm, diabetes, asthma, pulmonary, hepatic, hematological, renal, neurological, neuromuscular, and immune deficiency conditions. The representativity of individuals with one or more comorbidities, as well as their impact on survivability, is depicted in Figure 3.

**Table 1:** Characteristics of SARS-CoV-2–infected patients in the target cohort.

Characteristic				Value
**Numeric variables, mean (SD); range**				
	Age at notification (years)			48.3 (22.1); 0-105
	Onset to hospitalization (days)			1.1 (5.1); 0-169
**Categoric variables, n (%)**				
	**Gender**			
		Female	11,252 (54.64)	
		Male	9340 (45.36)	
	**Hospitalization**			
		Yes	16,651 (84.00)	
		No	3172 (16.00)	
	**ICU^a^ admission**			
		Yes	209 (7.75)	
		No	2488 (92.25)	
	**Respiratory support**			
		Oxygen therapy	276 (11.04)	
		Assisted ventilation	172 (6.88)	
		No support	2052 (82.08)	
	**Comorbidities**			
		Cancer	940 (4.56)	
		Cardiac disease	3025 (14.69)	
		Diabetes	2134 (10.36)	
		Immune deficiency	222 (1.08)	
		Renal disease	718 (3.49)	
		Liver disease	206 (1.00)	
		Lung disease	794 (3.86)	
		Chronic neurological disease	1087 (5.28)	
	**Mortality**			
		Yes	728 (7.45)	
		No	9050 (92.55)	

^a^ICU: intensive care unit.

### Hospitalization

**Figure 4:**
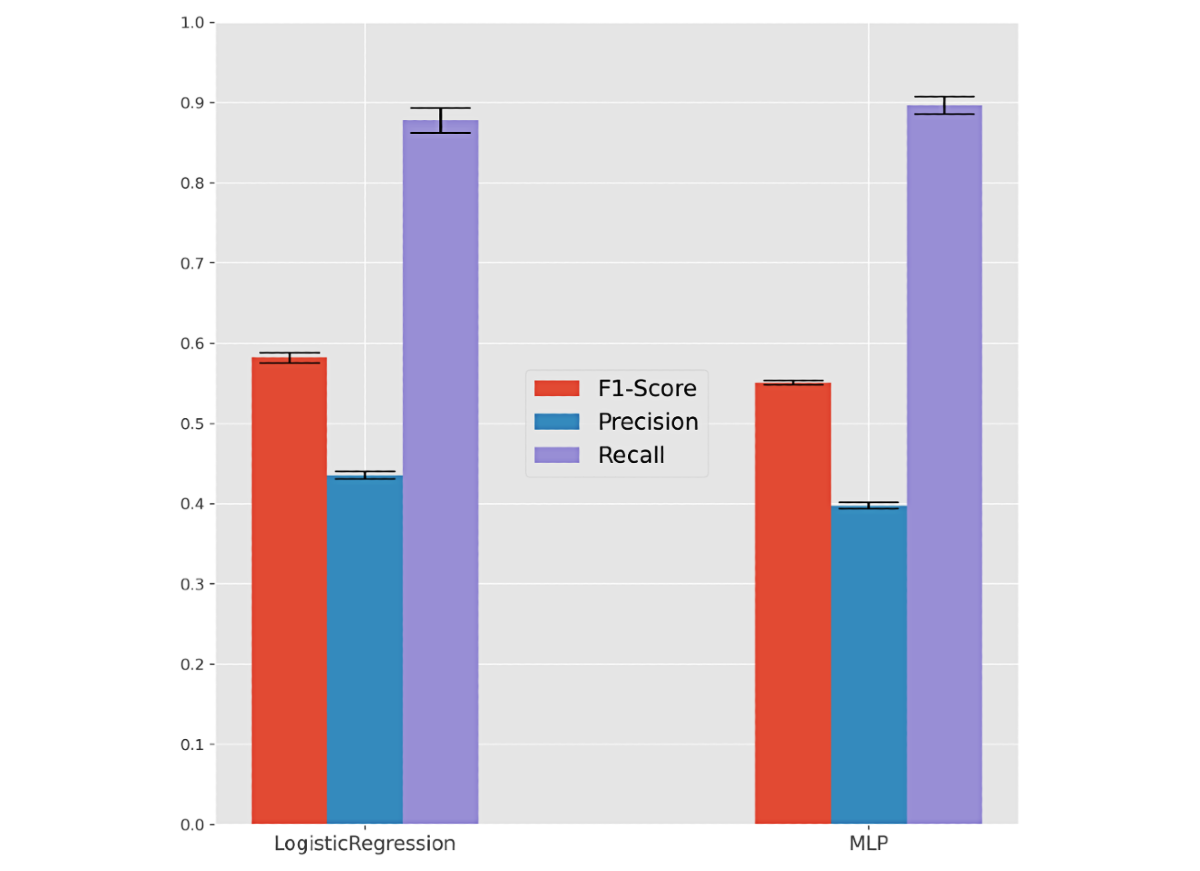
Predictability of hospitalizations for individuals testing SARS-CoV-2 positive. Recall, precision, and F1 for the best predictors in F1 (left) and recall-oriented (right) scores on the validation set after nested cross-validation. MPL: multilayer perceptron.

**Figure 5:**
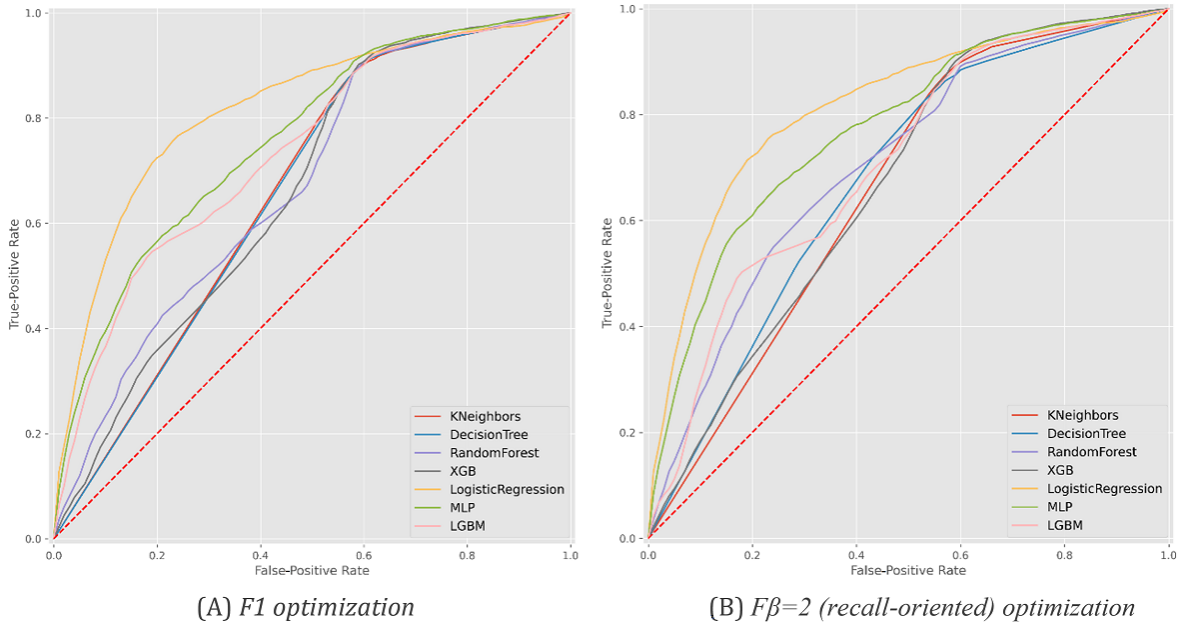
Receiver operating characteristic curves with the predictive behavior of the selected classifiers in asserting hospitalization needs at the time of SARS-CoV-2 testing. XGB: XGBoost, MLP: multilayer perceptron, LGBM: LightGBM.

Figure 4 and Table 2 provide results pertaining to the models’ ability to predict the need for individuals to be hospitalized once they are tested as SARS-CoV-2 positive given their (1) demographic group (age and gender) and (2) comorbidity factors. Comorbidity factors were categorized in accordance with the presence or absence of kidney, asthma, lung, cancer, neuromuscular, diabetes, HIV, cardiac, and pregnancy conditions. Nonhospitalized individuals without a clear outcome (recovery or death) were excluded from this analysis. Figure 5 provides the receiver operating characteristic curve per predictor for each optimization setting.

Generally, we observed that nearly 90% of hospitalization needs could be identified at the time of SARS-CoV-2 testing. This level of recall/sensitivity was observed at the expense of an approximate 55% precision, meaning that more than half of the predicted hospitalization needs were in fact observed. Logistic regression and MLP were the best-performing classification models according to F1-score and recall, respectively. Statistical superiority was verified for logistic regression but not MLP against peer models (at 𝞪=.05). These results provide empirical evidence toward the role of these predictors in supporting individual remote monitoring decisions.

**Table 2:** Predictability of hospitalizations per predictive model.

Model	F1 optimization, mean (SD)			Recall-oriented (F_*β*=2_) optimization, mean (SD)	
	F1-score	Recall	F1-score		Recall
KNN^a^	0.544 (0.007)	0.883 (0.020)	0.545 (0.005)		0.890 (0.017)
DT^b^	0.562 (0.030)	0.837 (0.069)	0.548 (0.004)		0.897 (0.007)
RF^c^	0.535 (0.010)	0.878 (0.016)	0.541 (0.005)		0.874 (0.029)
XGB^d^	0.546 (0.004)	0.897 (0.012)^e^	0.545 (0.004)		0.895 (0.011)
LR^f^	0.582 (0.006)^e^	0.878 (0.015)	0.583 (0.010)^e^		0.879 (0.015)
MLP^g^	0.549 (0.006)	0.892 (0.010)	0.551 (0.003)		0.897 (0.011)^e^
LGBM^h^	0.545 (0.005)	0.893 (0.013)	0.545 (0.005)		0.893 (0.016)

^a^KNN: k-nearest neighbors.

^b^DT: decision tree.

^c^RF: random forest.

^d^XGB: XGBoost.

^e^Best-performing models

^f^LR: logistic regression.

^g^MLP: multilayer perceptron.

^h^LGBM: LightGBM.

### ICU Admissions

**Figure 6:**
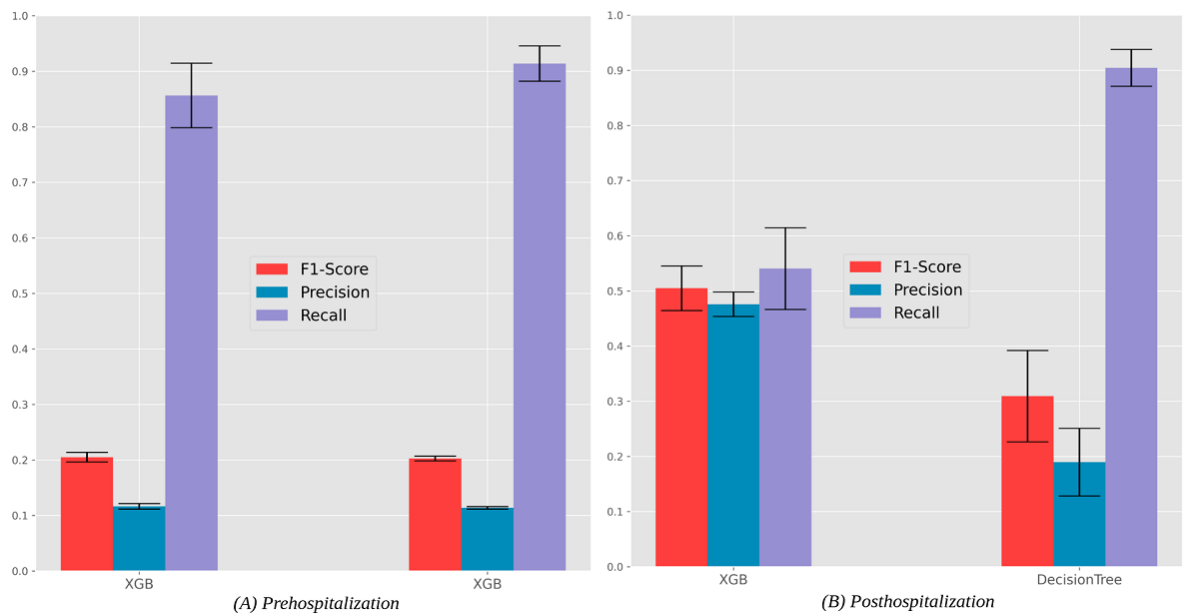
Predictability of intensive care unit admission. Results for the best F1 predictor (left) and recall-oriented predictor (right). XGB: XGBoost.

**Figure 7:**
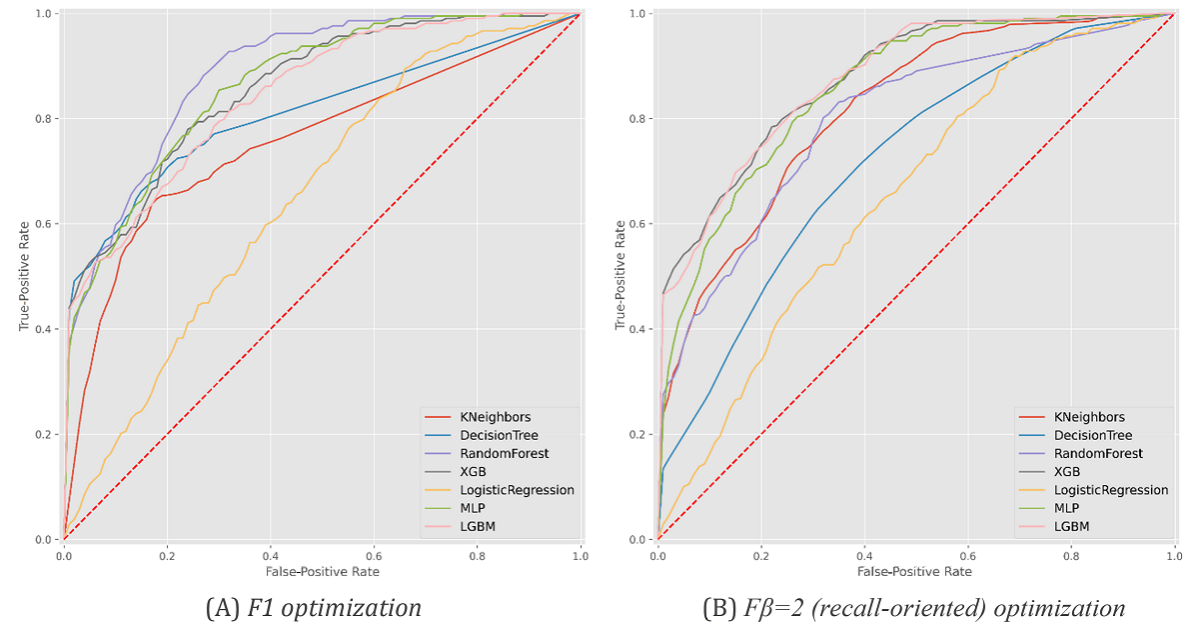
Receiver operating characteristic curves with the predictive behavior of the selected classifiers in predicting intensive care unit admission needs at the time of hospitalization. XGB: XGBoost, MLP: multilayer perceptron, LGBM: LightGBM.

Figures 6 and 7 and Table 3 assess the ability to anticipate intensive care needs for infected individuals at two stages: before hospitalization and after hospitalization. To this end, the proposed methodology was pursued considering demographic factors, comorbidity factors, and the time to hospitalization for hospitalized individuals. Individuals without a SARS-CoV-2–negative test result after infection were excluded.

**Table 3:** Predictability of intensive care unit admissions per predictive model.

Model			F1 optimization, mean (SD)			F_*β*=2_ (recall-oriented) optimization, mean (SD)	
		F1-score		Recall	F1-score		Recall
**At the time of hospitalization**							
	KNN^a^	0.428 (0.039)		0.574 (0.156)	0.369 (0.037)		0.741 (0.088)
	DT^b^	0.461 (0.016)		0.651 (0.056)	0.309 (0.083)		0.904 (0.033)^c^
	RF^d^	0.454 (0.027)		0.713 (0.088)^c^	0.382 (0.103)		0.794 (0.128)
	XGB^e^	0.505 (0.040)^c^		0.541 (0.074)	0.431 (0.040)		0.766 (0.084)
	LR^f^	0.250 (0.015)		0.622 (0.041)	0.248 (0.013)		0.651 (0.074)
	MLP^g^	0.449 (0.060)		0.703 (0.145)	0.410 (0.039)		0.818 (0.050)
	LGBM^h^	0.480 (0.023)		0.536 (0.048)	0.435 (0.025)^c^		0.770 (0.051)
**At the time of SARS-CoV-2 testing**							
	KNN	0.195 (0.016)		0.818 (0.090)	0.198 (0.007)		0.852 (0.039)
	DT	0.209 (0.012)		0.752 (0.135)	0.201 (0.007)		0.890 (0.036)
	RF	0.200 (0.008)		0.880 (0.038)	0.200 (0.008)		0.880 (0.044)
	XGB	0.205 (0.009)		0.857 (0.058)	0.203 (0.004)		0.914 (0.032)
	LR	0.200 (0.008)		0.847 (0.054)	0.201 (0.007)		0.880 (0.034)
	MLP	0.202 (0.006)		0.871 (0.049)	0.200 (0.008)		0.880 (0.037)
	LGBM	0.204 (0.012)		0.871 (0.074)	0.197 (0.012)		0.861 (0.066)

^a^KNN: k-nearest neighbors.

^b^DT: decision tree.

^c^Best-performing models.

^d^RF: random forest.

^e^XGB: XGBoost.

^f^LR: logistic regression.

^g^MLP: multilayer perceptron.

^h^LGBM: LightGBM.

The predictability of ICU needs is less satisfactory than hospitalization needs, particularly for the prehospitalization stage. We hypothesize that this difficulty was partially related to the smaller number of individuals with ICU internments, together with the presence of missing values associated with ICU internment needs for most individuals. Even though we can achieve recall levels over 90% with gradient boosting (XGBoost) in a posthospitalization setting, it comes at the cost of a considerably low precision (with one-third of predictions seen in practice). Still, the best-performing predictive models are suggested to support monitoring decisions at the hospital bedside, as their recall and specificity are considerably high.

### Respiratory Support

**Figure 8:**
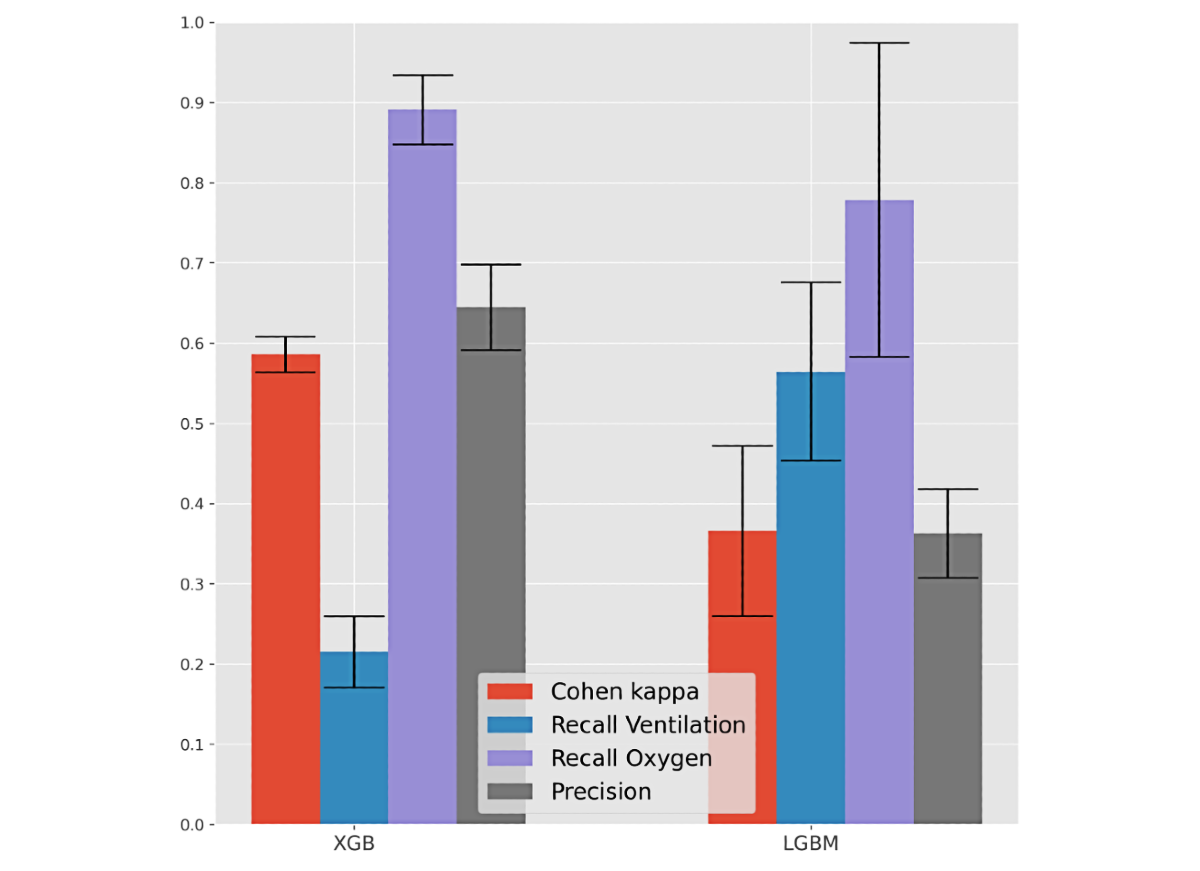
Predictability of respiratory support needs—assisted ventilation, oxygen therapy, and combined support—for hospitalized individuals with SARS-CoV-2. Performance of the best F1 predictor (right) and recall-oriented predictor (left) is shown. XGB: XGBoost, LGBM: LightGBM.

Figure 8 and Table 4 assess respiratory assistance needs for hospitalized individuals with SARS-CoV-2, considering three assistance modes: (1) ventilation support, (2) oxygen therapy, and (3) combined ventilation and oxygen therapies. Demographic, comorbidity, and time-to-hospitalization factors were used as input variables.

Individuals without a SARS-CoV-2–negative test result after infection were excluded from this analysis. As respiratory support is a multiclass variable, we considered a different performance evaluation by focusing on (1) the recall for each major class (ventilation, oxygen, and nonrequired support), (2) the precision of individuals with oxygen or ventilation assistance, and (3) the Cohen kappa coefficient.

XGBoost, LightGBM, and random forests attained a satisfactory identification of hospitalized individuals who may require respiratory support in the future, generally providing recalls for each assistance mode around 60% at the cost of a 40% precision. According to the conducted methodology, they are thus pinpointed as good candidates to support in-hospital care decisions.

**Table 4:** Predictability of respiratory needs per predictive model.

Model	Kappa optimization, kappa, mean (SD)	Recalls optimization average, kappa, mean (SD)
KNN^a^	0.324 (0.050)	0.162 (0.022)
DT^b^	0.708 (0.017)^c^	0.110 (0.126)
RF^d^	0.518 (0.013)	0.017 (0.014)
XGB^e^	0.586 (0.022)	0.043 (0.013)
LR^f^	0.080 (0.009)	0.070 (0.009)
MLP^g^	0.464 (0.046)	0.204 (0.164)
LGBM^h^	0.567 (0.037)	0.366 (0.106)^c^

^a^KNN: k-nearest neighbors.

^b^DT: decision tree.

^c^Best-performing models.

^d^RF: random forest.

^e^XGB: XGBoost.

^f^LR: logistic regression.

^g^MLP: multilayer perceptron.

^h^LGBM: LightGBM.

### Survivability (Outcome)

**Figure 9:**
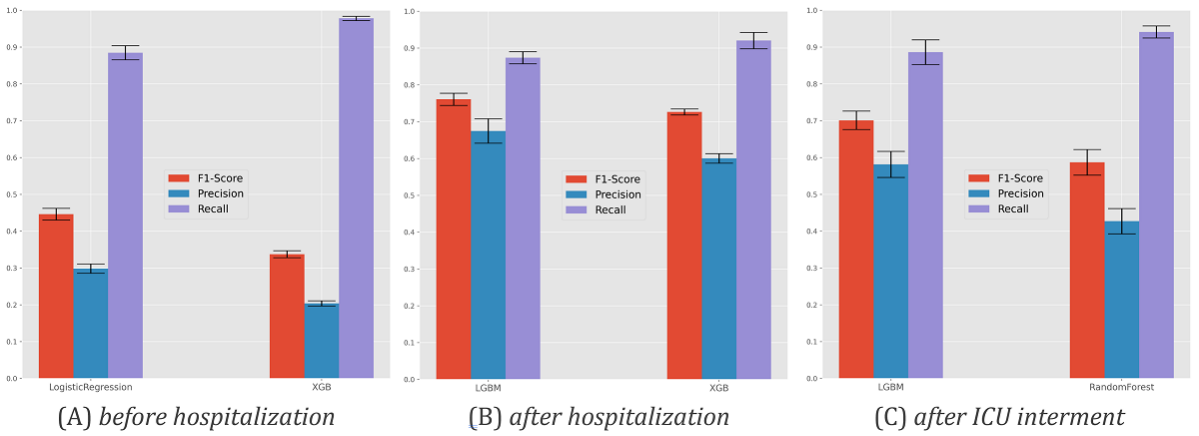
Predictability of the survivability (outcome) of infected individuals at 3 stages. Results for the best F1 predictor (left) and recall-optimized predictor (right) per stage are shown. XGB: XGBoost, LGBM: LightGBM.

**Figure 10:**
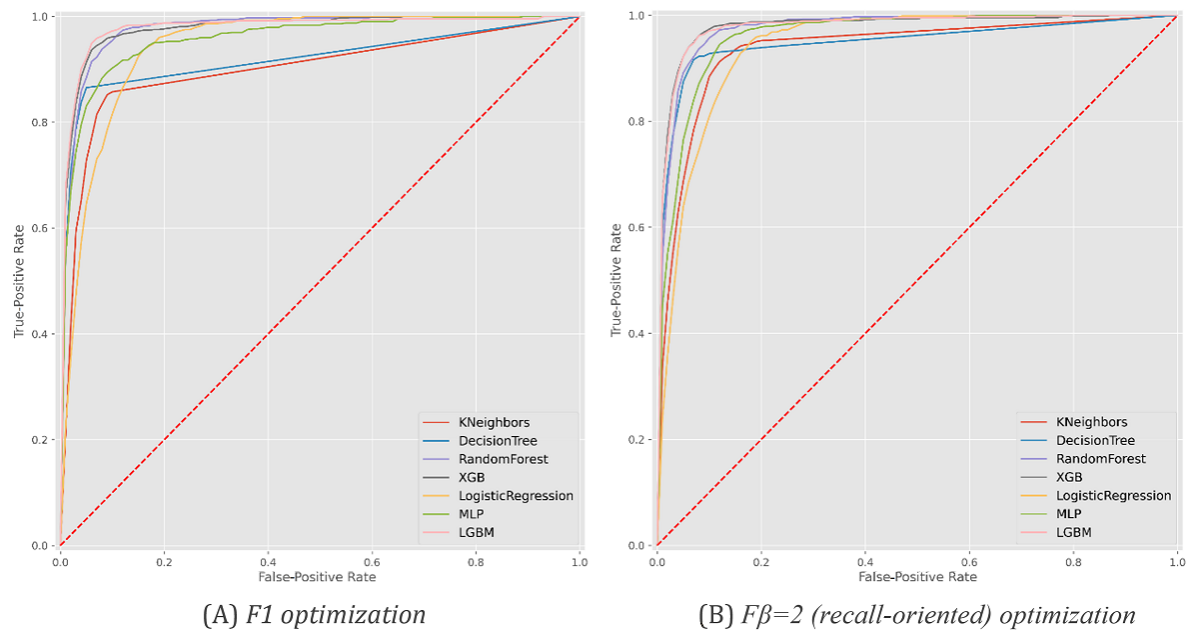
Receiver operating characteristic curves with the predictive behavior of the selected classifiers in asserting patient survivability at the time of hospitalization. XGB: XGBoost, MLP: multilayer perceptron, LGBM: LightGBM.

Finally, Figures 9 and 10 and Table 5 provide an analysis of the ability of the models to predict recovery-or-death outcomes for individuals with SARS-CoV-2 infection at three time points: (1) before hospitalization (at the time of testing), (2) after hospitalization, and (3) after ICU admission when applicable. To this end, we preserved the input variables and validation methodology (see *Methods* section) considered in previous scenarios.

Our results showed a high ability to identify death outcomes. However, at the SARS-CoV-2 testing stage, this comes at a cost of incorrectly classifying two-thirds of individuals susceptible to death. In the posthospitalization scenario, we achieved more balanced results, with both precision and recall around 75% using gradient boosting (XGBoost and LightGBM). The introduction of the intensive care variable hampered the results since it restricted the analysis of deaths to individuals with acute needs and dependent on continuous care instruments.

**Table 5:** Predictability of survivability per predictive model.

Model		F1 optimization, mean (SD)		F_*β*=2_ (recall-oriented) optimization, mean (SD)	
		F1-score	Recall	F1-score	Recall
**At the time of hospitalization**					
	KNN^a^	0.616 (0.035)	0.735 (0.048)	0.546 (0.021)	0.901 (0.030)
	DT^b^	0.707 (0.013)	0.864 (0.011)	0.673 (0.022)	0.908 (0.017)
	RF^c^	0.696 (0.030)	0.901 (0.030)	0.666 (0.021)	0.666 (0.021)
	XGB^d^	0.765 (0.025)	0.834 (0.042)	0.726 (0.008)	0.920 (0.022)
	LR^e^	0.492 (0.012)	0.909 (0.019)	0.476 (0.035)	0.916 (0.022)
	MLP^f^	0.681 (0.024)	0.824 (0.027)	0.569 (0.020)	0.922 (0.023)
	LGBM^g^	0.761 (0.017)	0.874 (0.016)	0.717 (0.036)	0.922 (0.021)
**At the time of intensive care unit admission**					
	KNN	0.582 (0.040)	0.740 (0.053)	0.527 (0.030)	0.885 (0.049)
	DT	0.652 (0.045)	0.879 (0.035)	0.638 (0.032)	0.922 (0.023)
	RF	0.630 (0.018)	0.908 (0.039)	0.587 (0.035)	0.941 (0.016)
	XGB	0.703 (0.035)	0.838 (0.068)	0.672 (0.021)	0.918 (0.051)
	LR	0.497 (0.018)	0.908 (0.031)	0.470 (0.049)	0.920 (0.028)
	MLP	0.633 (0.044)	0.790 (0.094)	0.529 (0.019)	0.935 (0.020)
	LGBM	0.701 (0.025)	0.886 (0.034)	0.672 (0.024)	0.915 (0.027)

^a^KNN: k-nearest neighbors.

^b^DT: decision tree.

^c^RF: random forest.

^d^XGB: XGBoost.

^e^LR: logistic regression.

^f^MLP: multilayer perceptron.

^g^LGBM: LightGBM.

### Determinants of Predictability

**Figure 11:**
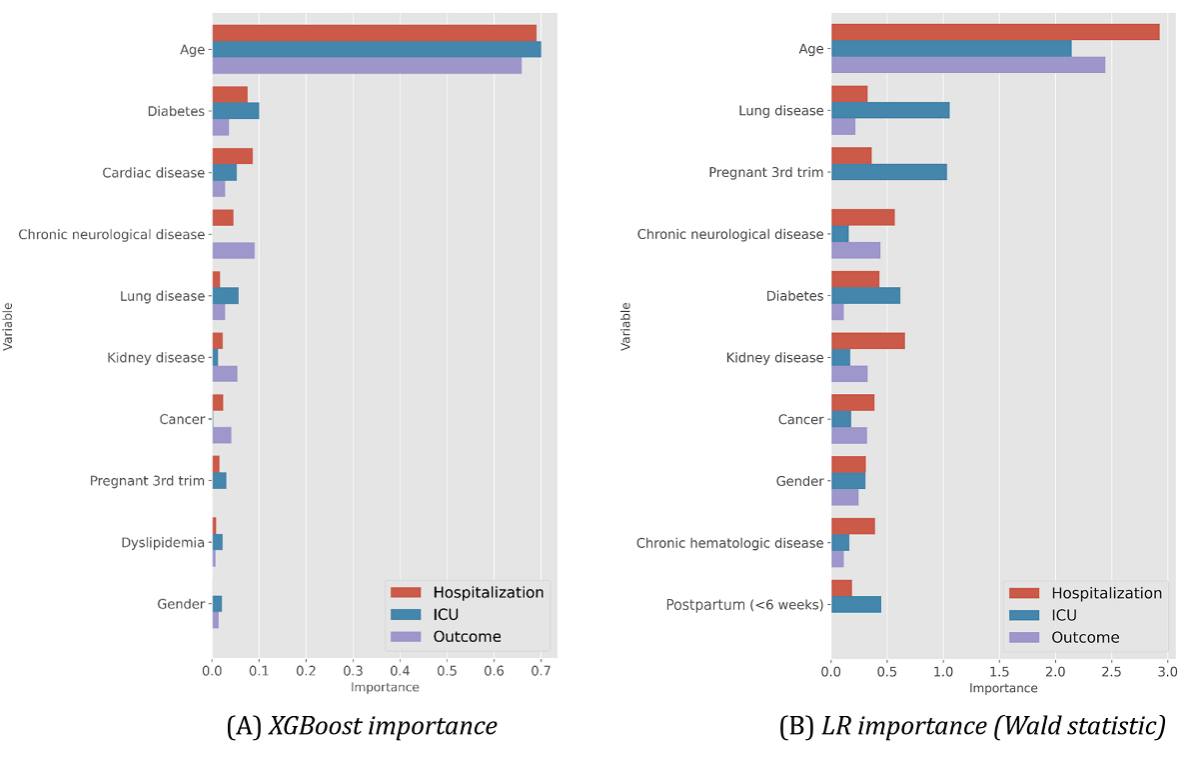
Top features and their importance for each target variable at the time of SARS-CoV-2 testing (prehospitalization). ICU: intensive care unit, LR: logistic regression.

**Figure 12:**
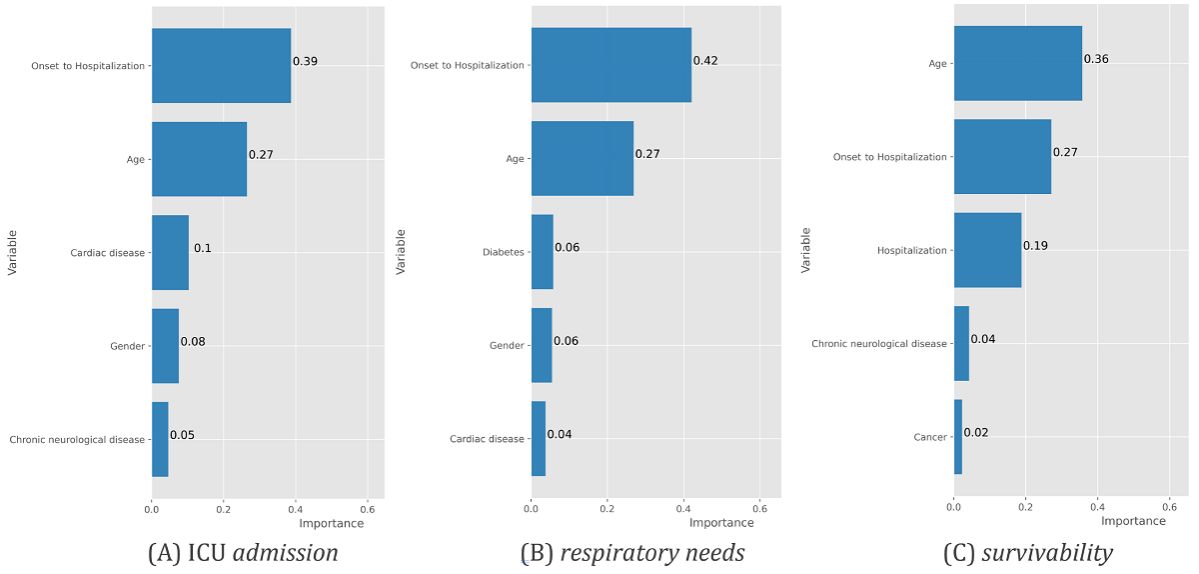
XGBoost top features and their importance for the different outcomes at posthospitalization. ICU: intensive care unit.

To assess the determinant factors underlying the achieved predictability levels, we first statistically tested the correlation between input and output variables using chi-square tests, ANOVA (analysis of variance), and their nonparametric counterparts, yielding results similar to those by Nogueira et al [33]. For a more in-depth understanding of the feature relevance for the assessed predictive models, Figures 11 and 12 illustrate the importance of the top features. To this end, we considered relevance outputs from gradient boosting (XGBoost) due to its competitively high performance across all outcomes, as well as the logistic regression for the hospitalization outcome by computing the Wald statistic to assess the significance of the coefficients for predictions.

We can observe that XGBoost distinguishes the relevance of different comorbidities for the target variables along each stage of the care process. In addition to the age variable, the onset period to hospitalization in days was also found to be a critical factor affecting the decisions (Figure 12). The high relevance of this variable consistently had top rank among associative models—XGBoost, random forests, and decision trees—pinpointing the importance of its collection for computer-aided predictions of ICU internment and respiratory needs.

**Figure 13:**
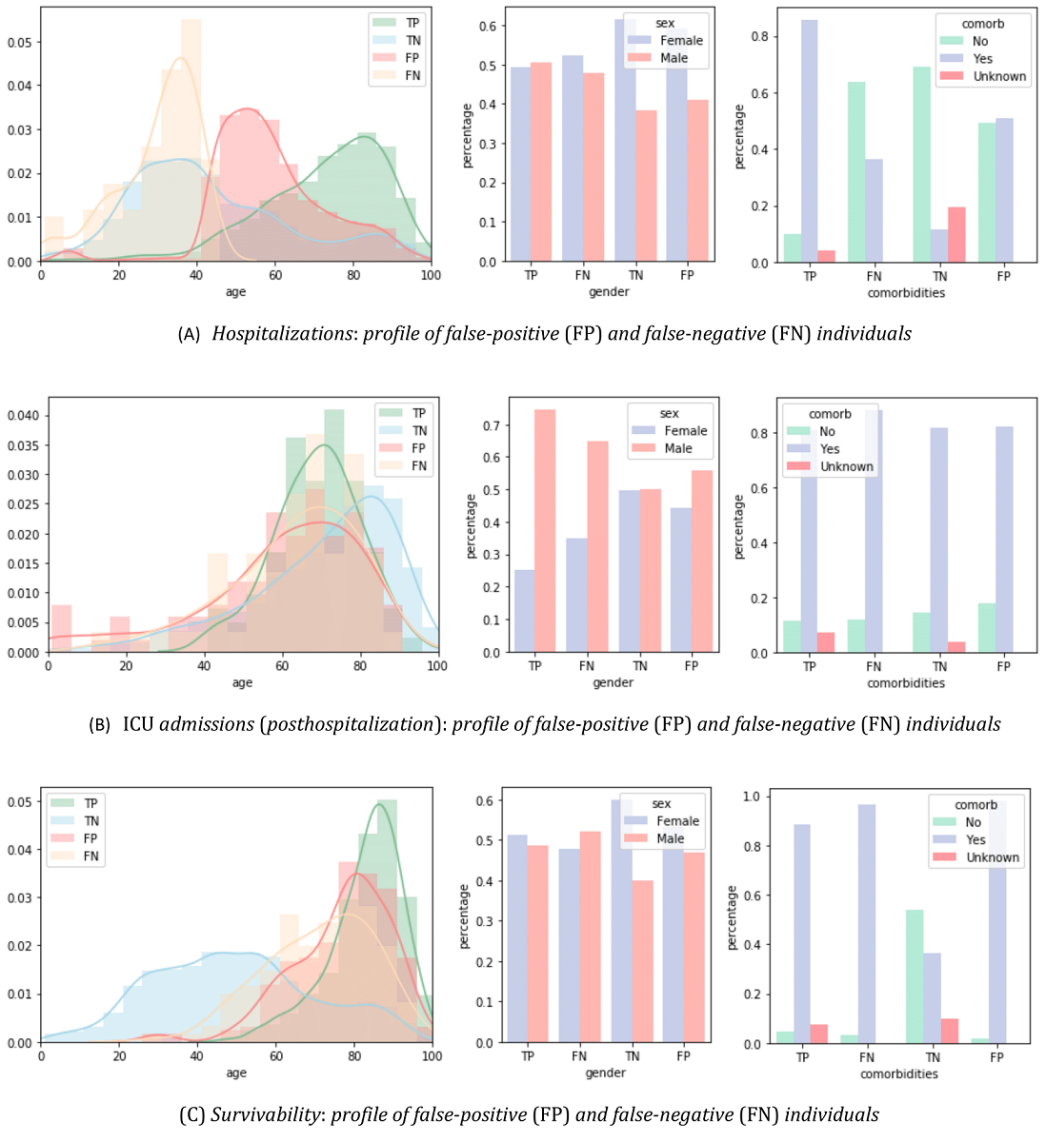
The characteristics of incorrectly predicted individuals with XGBoost. Particular attention should be paid to the differences between true-positive and false-negative individuals given their clinical relevance. ICU: intensive care unit.

Complementarily, Figure 13 offers additional insights into the target predictive tasks by plotting some of the characteristics of the correctly classified individuals against incorrectly classified individuals with XGBoost. Particular attention should be paid to the differences between true positives and false negatives, that is, to the individuals requiring care, in order to guarantee their timely and proper assistance. The susceptibility to false negatives is higher for individuals within the 40-60-year age category and without comorbidities.

### Clinical Decision Support System

**Figure 14:**
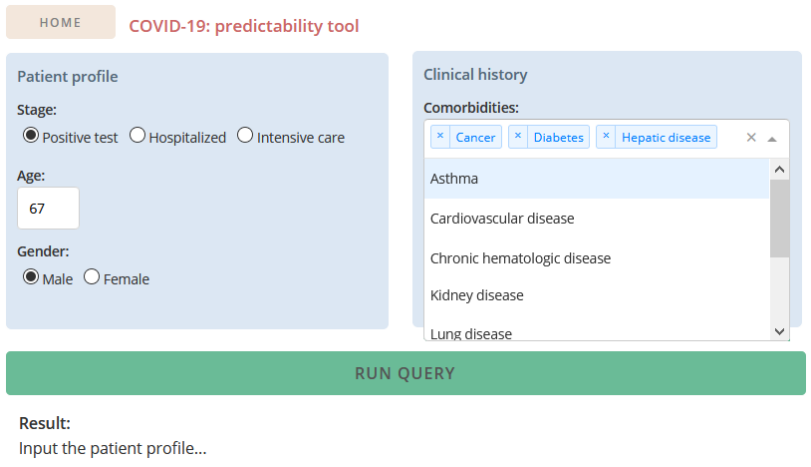
Snapshot of the provided clinical decision support system.

The learned predictive models based on simple variables (stage, age, gender, and comorbidities) have been made available to health care providers within a recommendation system with graphical facilities. The serialized predictive models are used for the efficient testing of individuals at the different stages of the care cycle (testing, hospitalization, ICU admission) for the different outcome variables (care needs) after inserting essential demographic and comorbidity features. The output provides a bounded statistic based on the estimation returned by the predictive models achieving better recall and F1-measure for each outcome variable. Figure 14 provides a visualization of the graphical interface.

The variables required for each outcome score calculation are usually available at hospitals, and the tool is easy to use. Although recommendations are provided within a statistical frame, the tool does not categorize the risk into low- or high-risk patients as clinical experts are more informed to approximate this risk. In addition, we advise caution for clinicians who intend to use this tool as a predictive guide, especially for survivability analysis. Clinicians must balance the predictions from this tool against their practical experience.

In collaboration with DGS, our predictors are expected to be provided within public hospitals and care contact centers of the Portuguese Health Service (Serviço Nacional de Saúde), particularly to support remote care monitoring decisions.

The decision support system is available as a software tool on GitHub [34].

## Discussion

### Principal Findings

This work offers a discussion on the predictability of hospitalization needs, ICU admissions, respiratory assistance, and survivability outcome in individuals infected with SARS-CoV-2 in Portugal as of June 30, 2020. A retrospective cohort with all confirmed COVID-19 cases since March, encompassing demographic and comorbidity variables, was considered as the target population in this study.

The results for the given cohort reveal that (1) over 75% of hospitalization needs can be identified at the time of SARS-CoV-2 testing (with >50% precision); (2) ICU needs are generally less predictable at both the pre- and posthospitalization stages in the given cohort; (3) respiratory assistance needs (including ventilation support, oxygen therapy, and combined ventilation-oxygen support) achieved recall levels above 60% (with >50% precision); (4) death risk along different stages (testing time, after hospitalization, and after ICU admission) had the highest degree of predictability.

The predictive models yielding better accuracy performance were associative classifiers, particularly XGBoost and RandomForests, neural networks with hyperparameterized architectures, and logistic regressors, with the optimal choice varying in accordance with the target variable and evaluation measure.

Publications on COVID-19 using machine learning models for different outcomes have been rapidly increasing. Gao et al [35] developed a model that includes the mortality risk prediction and reported an F1 ranging from 0.65 to 0.69 (κ=0.61-0.65), in line with our findings. Alternative studies [28,36,37] offer additional results for generalizing results and identifying population-specific differences. Yet, most of these studies do not comprehensively assess models’ performance or the cohort characteristics, impeding solid cross-population findings.

### Limitations

This study has some inherent shortcomings that should be noted: (1) the number of clinical variables for the outcomes of interest were limited (eg, BMI and clinical symptoms were missing); (2) further external validation of the selected models is required; and (3) although some inconsistencies (listed in the *Cohort Description* section) and missing/unknown entries in the original DGS data set were excluded, data acquisition problems may still persist and influence the outcomes of this work. The fully autonomous and parameter-free nature of the proposed computational approach/models allows it to be dynamically retrained with updated data.

### Concluding Remarks

In this work, we developed a web-based clinical decision support tool without biological variables as input that can be used by clinicians. The conducted work pinpoints the relevance of the proposed predictive models to aid medical decisions for the Portuguese population, including both remote monitoring and in-hospital care decisions. Predicting the most probable outcomes along the life cycle of a SARS-CoV-2–infected individual can identify patients who are expected to develop severe illness, thus optimizing the allocation of health care resources and supporting more vulnerable patients.

## Appendix

Multimedia Appendix 1 Optimized parameters for the best-performing classifiers (Figures 4, 6, 8, and 9).

## Abbreviations

ANOVA: analysis of variance
DGS: Direcção-Geral da Saúde
DT: decision tree
ICU: intensive care unit
KNN: k-nearest neighbors
LGBM: Light Gradient Boosting Machine
MLP: multilayer perceptron
SMOTE: Synthetic Minority Oversampling Technique
XGB: XGBoost
