# Supplementary figures and images for: Predictability of COVID-19 Hospitalizations, Intensive Care Unit Admissions, and Respiratory Assistance in Portugal: Longitudinal Cohort Study
Source: J Med Internet Res. 2021 Apr 28;23(4):e26075. doi: 10.2196/26075 (PMC8080965; doi:10.2196/26075)

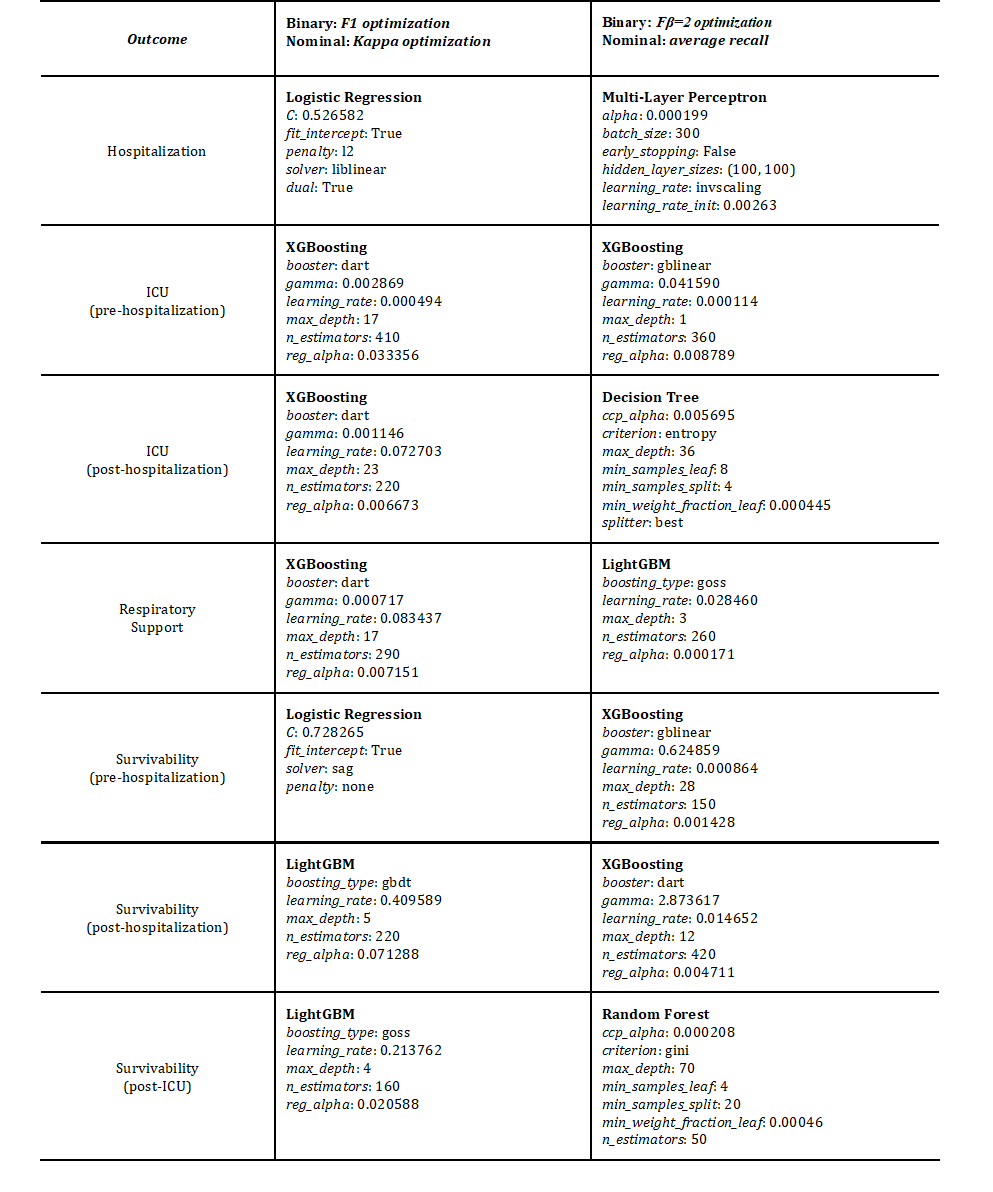

Supplement: Multimedia Appendix 1 [file jmir_v23i4e26075_app1.png]
